# Cariogenic Enamel Demineralization Prevention, Detection, and Management: A Literature Review

**DOI:** 10.1055/s-0045-1809179

**Published:** 2025-05-27

**Authors:** Rahaf Zawawi, Naif Almosa

**Affiliations:** 1 Independent Scholar, Private Sector, Specialty of Orthodontics and Dentofacial Orthopedics, Riyadh, Saudi Arabia; 2 Department of Pediatric Dentistry and Orthodontics, King Saud University, Riyadh, Saudi Arabia

**Keywords:** dental enamel, dental white spots, tooth demineralization, preventive dentistry

## Abstract

Dental caries is the most common oral disease, often resulting from complex and multifactorial interactions among cariogenic bacteria, fermentable carbohydrates, and host factors. Prevention is essential for reducing its incidence and can be achieved by maintaining a balanced diet and practicing good oral hygiene. Early detection of initial carious lesions, such as enamel demineralization, is essential for preventing progression and enabling effective management. Currently, there are various methods available for detecting dental caries, ranging from simple visual inspections to advanced imaging techniques, along with fluorescent and electrical detection methods, which are also instrumental in identifying lesions before they advance to more severe stages. Management strategies for enamel demineralization can vary from conservative methods, such as the use of remineralizing agents, to more invasive treatments. Herein, this article provides a comprehensive review of established and emerging approaches for preventing, detecting, and managing enamel demineralization for health care providers to take proactive steps toward improving oral health.

## Introduction

Dental caries is a complex disease that develops over time due to interaction between the host, cariogenic biofilm, and fermentable carbohydrates. 1 2 The initial carious lesion of enamel demineralization known as a white spot lesion (WSL), 1 2 that may result from persistent and episodic acid attacks, leading to the loss of enamel mineral and crystal density. 2

Enamel demineralization may present as softening of the outer surface due to the removal of the interprismatic substance, which creates a surface lesion. 3 4 This process is slow, and although some remineralization of the outermost layer may occur, deeper layers remain vulnerable to continuous loss of the interprismatic substance. 2 3 4 As a result, a subsurface lesion forms, giving the lesion a soft texture and causing it to appear rough, opaque, and milky-white when dried, with a fragile yet intact outer layer. 2

The normal enamel thickness varies depending on the tooth type and location. In deciduous teeth, enamel thickness ranges from 0.5 to 1.5 mm, while in permanent teeth, it ranges from 1 to 2.5 mm. 5 6 The initial enamel lesion is characterized by the penetration of a 10- to 100-μm thick intact surface layer with a subsurface core lesion and may be attributed to the lower crystal density at the enamel core compared to the outer layer. 7 8 The gaps created by mineral loss and microporosity are filled with air, leading to a difference in the refractive indices between the enamel and the air. 2 8 9 10 Additionally, the rougher surface caused by irregularities in the lesion and mineral loss leads to increased light scattering and changes in the refractive index, which in turn disrupts internal reflection and in a loss of surface translucency and shininess, causing the enamel to appear clinically opaque and dull. 9 10 It is important to note that higher enamel mineral content is directly associated with its translucency, thereby making the appearance of WSLs correlate with their mineral loss. 11 12

In these early stages, although the enamel remains intact, the lesion may become cavitated if subjected to a force. This progression can compromise the aesthetic appearance of the tooth or ultimately lead to dental cavitation. 7 8 Therefore, early detection and management of enamel demineralization are essential for successfully reversing lesions and minimizing their progression. With advancements in dental technology and materials, several modalities are available for detecting and managing enamel demineralization. Thus, understanding the methods for detecting WSLs and the available management approaches is crucial and clinically relevant for selecting the most effective conservative treatments. Herein, this present article reviews both the established and emerging methods for preventing, detecting, and managing enamel demineralization.

## Methods

To collect the necessary data for this literature review, searches were conducted across the PubMed, Cochrane Library, and Google Scholar databases using search terms “enamel demineralization,” “enamel demineralization detection,” “enamel demineralization prevention,” and “enamel demineralization management,” along with related terms such as “dentistry,” “white spot lesion,” “detection,” “prevention,” “management,” and “treatment” to ensure a comprehensive search.

The inclusion criteria for this review were as follows: (1) publications in English, (2) publication dates between January 1954 and January 2024, with an emphasis on more recent publications, while earlier studies were included for foundational scientific information, and (3) articles specifically addressing initial carious lesions (i.e., enamel demineralization) and their detection methods, prevention strategies, and management approaches.

**Fig. 1:**
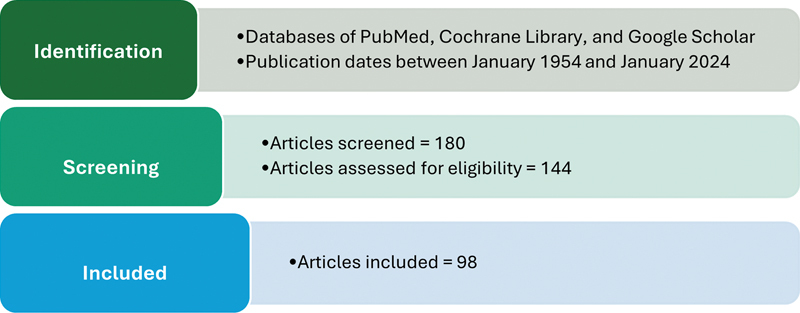
Flow chart summarizing the identification, selection, and screening process of the articles included in this review.

A total of 180 articles were reviewed, and 98 were found to meet the inclusion criteria and ultimately included in this review. Fig. 1 presents a flowchart that summarizes the identification, selection, and screening process of the articles included.

## Prevention of Enamel Demineralization

Prevention and remineralization approaches are often used interchangeably. This section discusses widely known prevention strategies, and approaches that are more impactful for remineralization are discussed in the management section.

### Diet

A cariogenic, or more accurately a potentially cariogenic diet can be defined as foods and drinks containing fermentable carbohydrates, which, in interaction with other factors, contribute to the occurrence of dental caries. 8 13 Acidogenic bacteria in the oral cavity can alter the environment to a pathogenic state in the presence of carbohydrates, causing a decrease in plaque pH below 5.5, which is conducive to enamel demineralization. 13 14 15 16

Regulating diet by reducing the intake of fermentable carbohydrates and incorporating probiotic foods can help balance oral bacteria and their acidic byproducts, thereby reducing the risk of caries initiation. 8 13 Additionally, a hard, fibrous diet structure can promote cleaner dental surfaces, in contrast to a sticky, carbohydrate-rich diet, which facilitates plaque accumulation. 8

The frequency of dietary intake also plays a significant role in caries prevention. More frequent consumption of food and drinks increases sugar intake and subsequently the chances of developing carious lesions, due to a more frequent pH drop in the plaque, which elevates the risk of demineralization. 8 13 14

A balanced diet not only helps prevent further lesion progression but also creates an environment conducive to remineralization. When combined with other preventive measures, such as proper oral hygiene and fluoride supplementation, a balanced diet can maximize the effectiveness of caries prevention. Even though the beneficial effects of diet in reducing the risk of enamel demineralization may make it a lesser contributing factor in the progression of lesions. 13

### Oral Hygiene

Maintaining proper oral hygiene through the use of fluoridated toothpaste and regular flossing is one of the most efficient and effective methods for preventing, halting, and remineralizing dental lesions. 8 14 Educating and motivating individuals to follow proper oral hygiene practices, such as brushing twice daily and flossing, along with scheduling regular professional checkups, are essential steps for preventing enamel demineralization and ensuring consistent dental health maintenance. 10 11 Good oral hygiene helps to mechanically disrupt dental plaque and alters its composition, promoting remineralization and breaking the cycle of demineralization. 3 Both manual and electric toothbrushes have been shown to be effective, with the choice between them generally depending on an individual's manual dexterity and personal preference. 10

### Fluoride Agent

Fluoride is essential for preventing dental carious lesions by inhibiting demineralization, primarily through its ability to bind strongly with minerals, particularly calcium and phosphate ions, which facilitates a net gain in minerals and helps counteract enamel loss. 13 17 However, the effectiveness of fluoride is constrained by the availability of actively accessible mineral ions, such as calcium (Ca ^2+^ ) and phosphate (PO _4_^3−^ ), which are necessary for its action. 13 17 Fortunately, saliva provides a natural reservoir of these mineral ions, which helps prevent demineralization and maintains the balance between the demineralization and remineralization cycles in the oral cavity. 13 17

Fluoride ions (F ^-^ ) penetrate the voids in enamel rods to bind with phosphate ions (PO _4_^3-^ ) and calcium ions (Ca ^2+^ ), 13 17 18 19 20 21 22 to replace hydroxyl groups (OH–) that naturally form the enamel apatite crystal lattice, resulting in the formation of fluorapatite (Ca _10_ (PO _4_ )6F _2_ ) rather than hydroxyapatite. 13 17 18 19 20 21 22 Fluorapatite is more stable within its crystal lattice compared to hydroxyapatite, as it interacts more strongly with calcium ions, thereby helping to retain these ions. 13 17 18 19 20 21 22

**Fig. 2:**
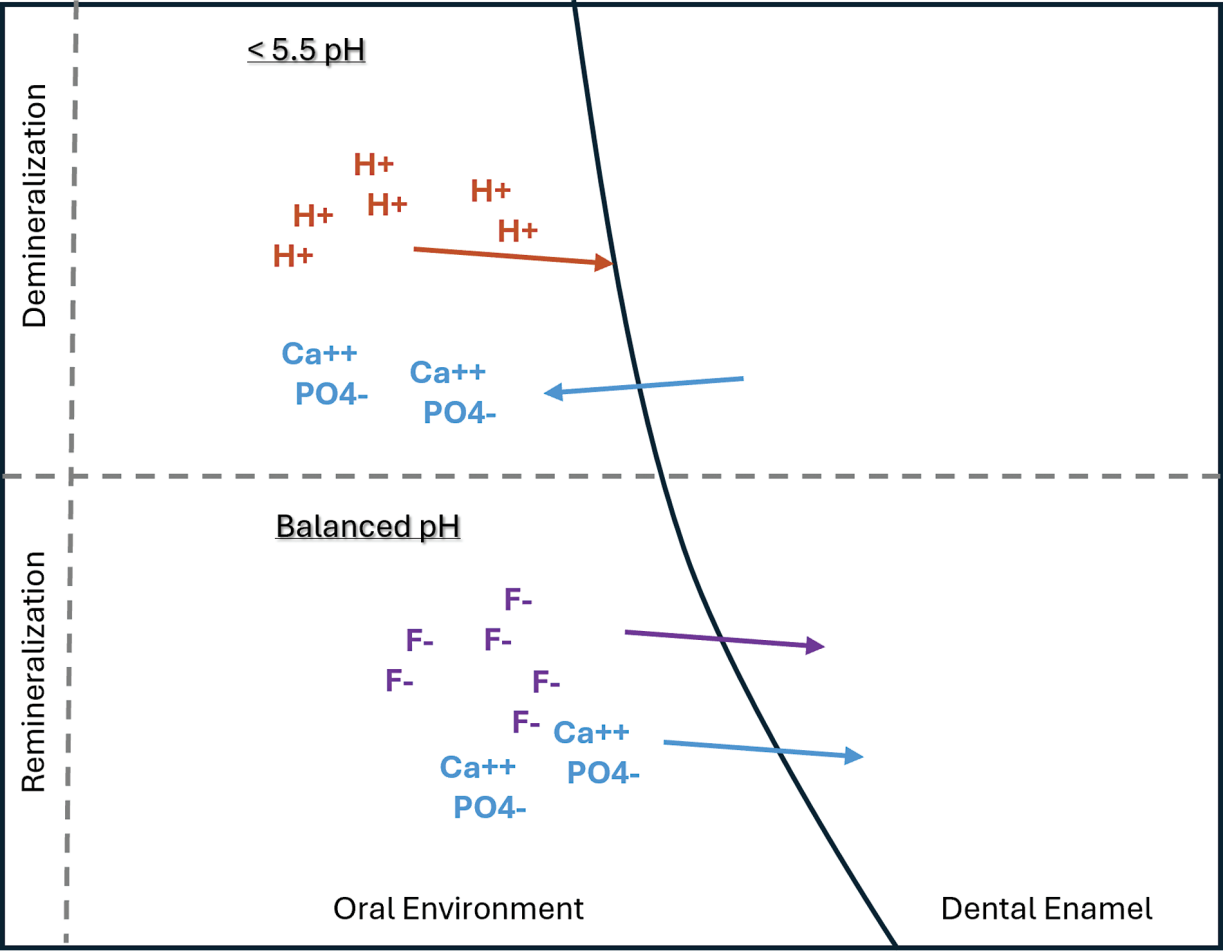
Enamel mineral exchange dynamicity. H + , hydrogen ions of acid attack; Ca + +, calcium ions; PO4-, phosphate ions; F-, fluoride ions.

This process, known as void theory, describes how fluoride fills the gaps and voids created by acid attacks within the hydroxyapatite crystals of enamel. As a result, fluorapatite has increased stability and lower solubility when exposed to acidic challenges, contributing to enamel protection and preventing further demineralization. 13 17 18 19 20 21 22 Fig. 2 illustrates the dynamic process of enamel, which can be detected using this approach.

There are several delivery methods for fluoride which include: (1) water fluoridation (at a low concentration of 0.7 ppm), which has been shown to prevent demineralization and enhance remineralization, (2) fluoridated milk, sugar, and salt (with limited evidence regarding their effect on caries prevention), (3) fluoride supplements (in the form of drops or tablets), which are effective in preventing dental caries but carry a risk of fluoride systemic toxicity if misused, (4) fluoridated toothpaste (at concentrations of 5,000 ppm), which has proven effective in promoting remineralization, particularly in permanent dentition), 13 23 (5) fluoridated mouth rinses and gels, which are effective in promoting remineralization and inhibiting demineralization in permanent dentition. 13 17 23 24 However, evidence supporting their effect on primary dentition is limited, and there is a risk of systemic toxicity and fluorosis if used improperly, 13 19 24 and (6) fluoride varnish, which has strong evidence supporting its effectiveness in promoting remineralization and inhibiting demineralization. 13 21 25 Its key advantage is that it can be applied with a high fluoride concentration to specific areas of risk, setting quickly before contamination can occur. 13 21 25

### Antimicrobial Agent

Chlorhexidine acts as both a bactericidal and bacteriostatic agent, providing a short-term preventive effect against the development of WSLs. 8 25 However, there is no direct association between its action and enamel remineralization. 8 25 A study by de Amorim et al found that combining fluoride varnish with chlorhexidine is more effective for the remineralization of early carious lesions compared to using fluoride varnish alone. 26

## Detection of Enamel Demineralization

The early detection of WSL is important for preventing irreversible loss of dental substrate and enabling timely intervention. 3 17 27 Various methods can be used to detect WSLs, ranging from traditional diagnostic techniques, such as visual examination and digital photographic evaluation, to more advanced technological approaches that assess the depth and extent of the lesions. These advanced methods include computer-aided analysis, transillumination, fluorescence, optical coherence tomography (OCT), phototherapy radiometry, modulated luminescence, electronic monitoring, and ultrasonic detection. 28

### Visual Examination

Visual inspection relies on the reflection of light from a clean and dried enamel surface to assess the presence of WSLs by observing changes in color and translucency. 2 8 It allows for the determination of lesion activity; a shiny and smooth surface indicates an inactive lesion, while an opaque, chalky, and rough surface suggests an active lesion. 29 Drying the tooth surface is essential because water has refractive index similar to healthy enamel, and when it fills the microporosity, this masks the reduced refractive index of the demineralized surface, making the changes less noticeable. 8

This method is the most commonly used due to its simplicity and cost-effectiveness, although it is subjective and lacks standardization. 28 To reduce inconsistencies in the detection of WSLs and the assessment of lesion extension, several indices for noncavitated lesions have been developed as diagnostic aids, such as the International Caries Detection and Assessment System (ICDAS), the Enamel Decalcification Index (1993), the WSLs Index by Gorelick et al (1982), and the Nyvad Index. 29 30 31 32 For instance, the ICDAS coding scale ranges from sound tooth surfaces, with no evidence of caries after prolonged air drying, to distinct cavities with visible dentin. 33 34

### Digital Photographs Evaluation

Digital photographs are primarily used for visual assessment through captured images. While this method offers the advantage of remote assessment, it is technique-sensitive and requires expensive, high-quality equipment to ensure proper photo quality and standardization. 8 Nevertheless, lesion detection and severity scoring based on digital photographs have been demonstrated to be both reliable and valid methods. 35

### Computer-Aided Analysis

The integration of digital advancements into the dental field has been increasing, particularly for its less invasive nature and the reliable, reproducible outcomes it provides. In addition, computerized image processing software allows analysis of area and pixel values of predefined lesion margins in magnified grayscale digital photographs, providing information about the size, shape, and color luminance intensity of WSLs. 36 37

Deep learning is another emerging method that uses an artificial model built on layers of self-learning algorithms to assess light and grayscale intensity for detecting the presence and stage of a lesion based on a stored database. 38 39 Although it has the advantages of being simple and capable of being performed using smartphone images incorporated into the software, it requires standardized lighting and additional training. 37 38 39 40

### Transillumination

**Fig. 3:**
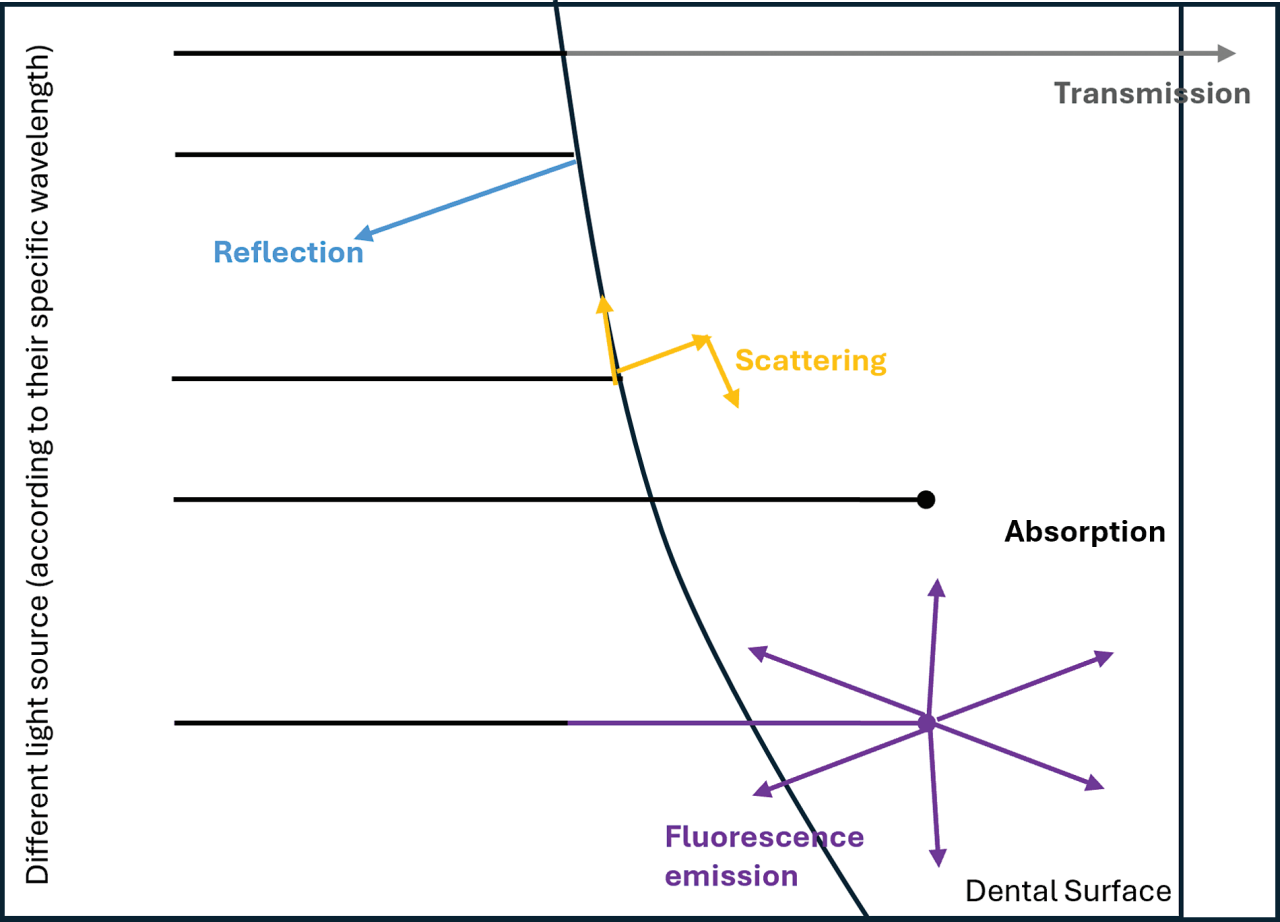
Different light interaction with enamel surface.

Dental surfaces interact with light in various ways, depending on the type of light, its wavelength, and the physical properties of the dental surface, which can be influenced by its chemical composition and physical arrangement. 13 Fig. 3 illustrates how light interacts with enamel surfaces at specific wavelengths, enabling the use of light for detecting enamel demineralization.

The transillumination technique relies on the difference in light transmission between intact and affected enamel, with the transmission coefficient being higher for intact enamel. 8 Fiber optic light transillumination (FOTI) utilizes light transmission to pass through the enamel surface. 8 When assessing WSLs with FOTI, the lesions appear darker than the intact enamel; however, this method has limited ability to detect lesion depth. 40 41

Digital FOTI (DFOTI) combines the light transmission capabilities of FOTI with a camera, using nonionizing infrared radiation, which is then processed and analyzed using integrated software for lesion monitoring. 2 8 DFOTI can detect early-stage lesions within 2 weeks, based on the light power of the fiber optic device; however, it cannot differentiate lesions' depth. 8 42

Near-infrared light transillumination takes advantage of the contrast difference between mineralized and demineralized surfaces by subjecting near-infrared light on the lingual and buccal surfaces, allowing the light to pass through the gingiva and scatter toward the tooth surface. 8 43 The scattered light is then captured by a charge-coupled device sensor and converted into an image, providing enhanced light data for further analysis. 42

### Fluorescence

This method is based on the autofluorescence phenomenon, in which enamel structures can emit light after absorbing it, and the emission may differ based on the amount of mineral lost ( Fig. 3 ). 44 Quantitative light-induced fluorescence (QLF) works by directing intense light through a blue filter onto the tooth surface and analyzing the resulting fluorescence using device software. 43 45 The QLF tool shows lesions as darker areas, indicating reduced fluorescence (normal enamel fluorescence occurs at a 488-nm wavelength), with data reflecting the lesion's size and density. 46 47 48 Moreover, QLF can detect red fluorescence from microorganisms, enabling the assessment of oral hygiene and the identification of surfaces covered by bacteria. 46

Laser fluorescence (LF) is utilized in DIAGNOdent, which uses infrared light directed onto the tooth surface through an optical fiber to collect fluorescence readings. 45 47 The readings correlate directly with lesion depth and are known for their high sensitivity despite low specificity. 45 46 Moreover, DIAGNOdent has also shown reasonable sensitivity when compared to the clinical index ICDAS-II. 49

A modified version of DIAGNOdent, called the DIAGNOdent pen, operates on the same principle but is designed with different tips for various applications. 48 50 Lussi and Hellwig found that both the DIAGNOdent and DIAGNOdent pen had similar abilities in detecting occlusal caries. 50 The SoproLife camera, a more recent development, also uses LF and offers the advantage of high magnification for intraoral imaging. 46 50

### Dye

Caries detection using dyes is widely recognized for identifying dentin caries. 51 52 However, when applied to enamel, these nonspecific dyes can stain food debris, the enamel pellicle, and other organic matter that may be trapped in significant amounts within occlusal fissures, which can lead to irreversible staining, and is clinically unacceptable. 13 51 52 Additionally, there are concerns about the potential for false positive or false negative results when detecting initial demineralization lesions. 52

To address these limitations, dyes are often used in combination with other demineralization detection methods to enhance diagnostic accuracy, such as in QLF. 13 51 52 The intensity of fluorescence from these dyes correlates with mineral loss, but, like dentinal caries detector dyes, they are specific only to demineralization. 51 52 Dyes such as Procion dyes, Calcein dye, fluorescent dye, and Brilliant Blue dye fill the porous structure of infected enamel without causing physical or chemical reactions, significantly increasing the contrast in images of artificial incipient lesions. 48 51 53 However, their lack of specificity can result in false positive readings. 48

The new patented dye, called BlueCheck (European Patent No. 2 547 311 B1), has been developed for detecting early demineralization by reversibly staining porous apatite. 54 The active component of this dye is a hydroxyapatite-binding protein, specifically hemoglobin, which strongly binds to porous apatite. 54 This deep blue Amido Black dye stains demineralized areas of porous enamel, causing them to appear dark blue. 54 As a promising, noninvasive tool, it requires no additional auxiliary materials; however, clinical studies are needed to confirm the *in vitro* findings. 54

### Optical Coherence Tomography

OCT utilizes infrared technology to recombine scattered light from the dental structure into two-dimensional and three-dimensional images, providing a cross- sectional images of the internal tooth structure based on demineralized enamel surface porosity and light depolarization. 45 55 56 OCT delivers both quantitative and qualitative data for early lesion detection, lesion depth assessment, detection of cracks and fractures, and evaluation of restorative marginal adaptation. 46 55 It can be applied in both *in vitro* and *in vivo* settings. 46 55 However, the sensitivity of OCT decreases as the lesion extends deeper into the dentin due to the similar light scattering properties of demineralized and normal dentin, and has depth limits of approximately 1 to 2 mm. 54 56

Polarization-sensitive OCT (PS-OCT) is a recent modification that enhances the OCT mechanism by capturing scattered light in the polarization state of the tooth. 56 57 The use of polarization in PS-OCT reduces the high light reflectivity from the tooth surface, resulting in clearer readings, more accurate measurements, and improved lesion monitoring, which reflects the demineralization and remineralization status. 56 58

### Photothermal Radiometry and Modulated Luminescence

Photothermal radiometry and modulated luminescence (PTR–LUM) utilize high-energy laser applications on the tooth surface, generating various thermal energy waves that are converted into optical readings for lesion detection and monitoring. 59 The commercially available Canary System has been shown to possess higher sensitivity than visual examination, intraoral radiographs, cone-beam computed tomography, and DIAGNOdent. 59

### Electronic Monitor

The electronic caries monitor (ECM) measures the altered electrical conductivity of enamel, which is influenced by enamel porosity filled with saliva and fluid, as well as temperature changes. 8 46 58 59 60 ECM is particularly recommended for smooth and proximal surfaces and has been found to have reasonable accuracy in detecting WSLs. 8 46 61 Another modern tool, CarieScan, utilizes multiple electrical frequencies (as opposed to the single frequency used by the ECM) to differentiate between healthy and demineralized tooth surfaces, offering very high detection accuracy and reliability. 8 60 61

### Ultrasonic/Ultrasound Detector

The ultrasound caries detector uses acoustic frequency waves and their duration, which correlate with changes in mineral content, to assess lesion depth with high sensitivity and specificity. 14 41 62

## Management of Enamel Demineralization

WSLs naturally remineralize at a slow rate, particularly when obstacles to cleansing, such as fixed orthodontic appliances, are removed. 22 Various prevention modalities, including diet, oral hygiene, fluoride agents, other remineralization agents, antimicrobial agents, and oral probiotics, play a role in preventing and managing initial enamel demineralization, as discussed in the prevention section. Other management approaches, such as dental bleaching, microabrasion, ozone therapy, and laser treatment, are also available.

### Fluoride

Fluoride has several key benefits, including its ability to inhibit demineralization, promote remineralization, and strengthen enamel, making it the primary choice for enhancing the remineralization process. A low-level fluoride concentration of 50 ppm is particularly effective, as it acts as a catalyst in the mineral exchange cycle. 13 17 18 The recommendation for low fluoride dosage is based on its limited solubility, which restricts its ability to penetrate deep lesions (with a depth limit of 50 μm), since high concentrations of fluoride can remineralize the outer layers of the enamel, leaving the deeper, demineralized areas unaffected. 17 Linton found that 50 ppm fluoride concentration was more effective than a 225-ppm fluoride concentration. 19

A meta-analysis by Yeoh et al concluded that removing fixed orthodontic appliances while maintaining proper toothbrushing with fluoridated toothpaste can effectively reduce postorthodontic WSLs. 20 Moreover, fluoride agents have been shown to improve the remineralization status, although the results are heterogeneous, favoring the use of low-concentration fluoride. 20 21 Willmot found that the natural remineralization of WSLs over 6 months can reduce lesion size similarly to the use of low-fluoride mouth rinse formulations. 22

Since the remineralization capacity of fluoride can be enhanced when combined with calcium ions, 17 remineralization agents typically consist of minerals, primarily calcium and phosphate, with or without proteins. 17

### Other Remineralization Agents

Amorphous calcium phosphate (ACP) utilizes a two-phase application, preventing the interaction between calcium and phosphate and ensuring they remain active until application. 2 ACP has been found to have a similar effect to fluoride in improving the color of WSLs. 25 Casein, a naturally occurring mineral-protein combination, helps prevent and reverse caries lesions. 17 However, the effective amount needed to produce a toothpaste formulation creates an unpleasant taste. 17

A complex of casein phosphopeptides-ACP (CPP-ACP) is a commercially available formula that features free mineral ions, facilitating easier mineral transfer to the enamel. 63 A study by Reynolds et al on animal teeth found that a 1.0% concentration of CPP-ACP reduced WSLs by 55%. 64 Recently, CPP-ACP was confirmed to enhance the enamel remineralization process on human teeth. 63

Sodium calcium phosphosilicate (bioactive glass [BAG]) works by releasing mineral ions into the saliva, which trigger enamel remineralization. A key advantage of BAG is the continuous release of ions after the initial application. 2 An *in vitro* study demonstrated that BAG had a higher potential to remineralize WSLs compared to CPP-ACP. 64 However, randomized clinical studies are needed to draw clear conclusions regarding BAG efficiency in treating WSLs.

The calcium carbonate carrier (SensiStat) contains calcium carbonate particles carried through the amino acid complex of arginine bicarbonate, which helps particles adhere to and dissolve on the dental surface, releasing calcium ions to initiate remineralization. 65 Further studies are required to confirm its remineralization properties.

Di-calcium phosphate di-hydrate (DCPD) increases calcium ion levels when incorporated into toothpaste, particularly in its interaction with fluoride. DCPD has been shown to delay demineralization and effectively reduce WSL size, surface porosity, and remineralize deeper layers reaching the dentin. 66 67 68 69

Alpha tricalcium phosphate (ATCP) and trimetaphosphate ion (TMP) both operate on the principle of providing a medium rich in calcium ions for remineralization. 2 70 Current data indicate that ATCP has significant potential to remineralize enamel and rebuild porous structures. 71 Moreover, when combined with fluoride-based products, ATCP exhibits higher remineralization potential than CPP-ACP-based products. 72 TMP contributes a high mineral content to the enamel surface to enhance the remineralization process, and this effect can be further amplified when combined with fluoride products. 70 73

Nanohydroxyapatite (nHA) uses nano-sized hydroxyapatite particles that have a high affinity for incorporation within enamel rods and porosities. nHA particles are more biocompatible with higher bioactivity and mechanical properties than regular hydroxyapatite. 74 75 Studies have concluded that 10% nHA toothpaste is an effective repair material for initial carious lesions, with similar or even superior potential to fluoride-containing toothpaste, improving remineralization capacity and reducing WSL depth and size. 2 74 75 76 77 78 79 nHA is a promising agent due to its high biocompatibility and remineralization capacity, but further studies are required to establish strong clinical evidence. 79

### Oral Probiotic

Probiotics refer to living microbes that are beneficial to the host, with specific strains used in the prevention and management of dental caries, including *Lactobacillus rhamnosus* GG, *Lactobacillus casei* , *Lactobacillus plantarum* , *Lactobacillus reuteri* , and *Bifidobacterium* species. 80 81 The role of probiotics in carious lesions is centered around reducing acidogenic bacteria and balancing the pH in the oral environment. 80 Probiotics can be administered through diet, tablets, or topical agents, such as toothpaste. Studies evaluating the probiotic effects on carious lesion remineralization have shown that probiotics have reasonable potential for reducing lesion size and depth. 82 83 However, further evidence is needed to conclusively establish the role of oral probiotics in enamel remineralization.

### Dental Bleaching

Tooth whitening or brightening helps camouflage lesions as areas of intact enamel become lighter than WSL. 8 However, bleaching does not alter the chemical or mechanical properties of the enamel. 6 84 85 Gizani et al reported in a systematic review that there is weak evidence for the efficacy of bleaching in improving the aesthetic appearance of WSLs. 86

### Microabrasion

Microabrasion is a minimally invasive treatment that uses either acid or abrasive particles, applied with a rubber cup and gentle pressure. 6 8 87 The primary indication for this technique is the removal of a thin layer of enamel, especially from porous and irregular surfaces, and the elimination of intrinsic stains that adhere to the enamel, 8 17 87 leading to a smoother surface by removing the porous outer layer of enamel. 8 17 When performed correctly, microabrasion should not cause significant enamel loss, pulp irritation, or damage to periodontal tissue, while also improving the aesthetic appearance of the teeth. 87

Microabrasion is particularly effective in reducing the size of lesions and enhancing their appearance, especially for lesions no deeper than 0.2 to 0.3 mm, 63 87 88 89 which makes it most suitable for treating shallow lesions. 63 87 88 89 Additionally, the results of microabrasion typically last up to 12 months, which necessitates long-term maintenance. 8 To achieve the best aesthetic outcome, it is recommended to begin with a remineralization agent, and if necessary, combine microabrasion with resin infiltration, particularly for deeper lesions. 87

### Resin Infiltration

This technique involves filling enamel microporosities with low-viscosity resin instead of completely removing the lesion surface. 8 Studies have shown that resin infiltration is highly effective in improving aesthetics compared to microabrasion. 86 88 The resin-enamel interface enhances surface integrity, making the enamel more resistant to acid attacks. 8

### Ozone

Ozone disinfection can effectively reduce the number of acidogenic bacteria and promote a more balanced environment conducive to remineralization due to its strong oxidation properties, 90 91 which allows it to oxidize bacteria and act as a bactericide. 13 However, while ozone provides a significant immediate bactericidal effect, it does not offer long-term results, as bacteria can recolonize over time. 13 92 Although ozone does not have a lethal effect on humans, these findings underscore the need for clear guidelines and scientific evidence before its broader application. 13 93

### Laser

The use of lasers in dental treatments can modify the tooth surface by promoting the recrystallization of superficial hydroxyapatite crystals, which increases the mineral's solubility and absorption, while also inducing thermal changes in the enamel's organic matrix. 94 95 These effects contribute to a reduction in enamel permeability and solubility, enhanced resistance to dissolution, decreased subsurface demineralization, and improvements in the aesthetic appearance of WSLs. 17 94 95

Studies have shown that combining lasers with fluoride agents does not significantly enhance the fluoride efficacy in reducing the extent of WSLs. 96 Ahrari et al found that sodium fluoride, whether used alone or in combination with a laser, was the most effective method for increasing WSL hardness, but this combination did not provide an additional benefit in terms of remineralization. 97 These findings suggest that further research is required to better understand the laser's effect on enamel and its potential role in remineralization.

### Plasma Treatment

Plasma, a biocompatible gaseous medium, exhibited bactericidal properties and can penetrate irregular surfaces. Cold plasma treatment has been shown to enhance the remineralization capacity and increase enamel hardness, especially when combined with bioactive bioglass materials, which are paste-formed substances rich in calcium phosphate. 64 98

## Discussion

Dental caries is the most common oral disease, making prevention and early detection/management essential for overall oral health. 1 2 7 8 This article reviews established and emerging methods for preventing, detecting, and managing enamel demineralization.

Prevention is essential for maintaining enamel integrity. One of the key strategies is to ensure proper oral hygiene, which includes toothbrushing twice a day with fluoridated toothpaste and flossing regularly. 8 10 11 These practices can help preserve enamel integrity, prevent demineralization, and promote remineralization. 10 11 Regular professional dental checkups are also recommended. 10 11 These visits allow for the application of topical fluoride when necessary and enable early detection of dental issues, which can boost motivation for good oral care and provide essential education on oral health.

Maintaining a healthy diet that is low in fermentable carbohydrates is important as well, although its impact is less significant when proper oral hygiene is practiced. 8 13 Additionally, antimicrobial agents can be beneficial in reducing bacterial levels in the mouth, but their use should be customized to meet individual oral health needs. 8 25 Ultimately, effective oral hygiene is the best defense against dental caries.

Early detection is essential for minimally invasive management, and this review covers several detection approaches. 3 17 27 When comparing digital photo assessment and dyes, visual inspection remains the first approach as it does not require any additional equipment or costs. Although digital photos and computer-aided analysis can be effective, they necessitate training and are sensitive to technique. 8 37 38 39 40 The innovative dye (BlueCheck) is promising, but further studies are needed to establish its effectiveness. 54 Current dyes tend to be more beneficial when used in conjunction with other tools. 13 48 51 52 53

Various transillumination and fluorescent tools offer reasonable sensitivity, but their limitations include restricted detection areas and limitations in the type of assessment—such as lesion surface width or depth—along with the need for extra equipment and associated costs. Among more advanced detection methods, PTR-LUM has demonstrated higher accuracy. Nevertheless, additional evidence is required to compare it to ECM and ultrasonic/ultrasound detectors. In summary, while visual inspection remains the gold standard, further research is necessary to compare the sensitivity and specificity of these different detection tools.

Management of enamel demineralization depends on the severity of the lesion, which can be assessed by surface width, depth, and color difference. Some approaches support natural remineralization, emphasizing the importance of maintaining good oral hygiene and utilizing low-dose minerals to prevent oversaturation during mineral exchange. 22 Others prefer the application of low fluoride as first-line management, which aims to remineralize lesions. 17 18 19 Research has shown that fluoride is absorbed more effectively in conjunction with other minerals, indicating that alternative remineralizing agents can be beneficial. 17 18 19 20 64 65 67 68 69 70

Several studies indicate that nHA may have greater remineralization potential than fluoride, but the durability of its solubility resistance remains uncertain. 2 74 75 76 77 78 79 Additionally, approaches that influence the composition or quantity of oral flora, such as probiotics, ozone, and plasma treatments, are expected to have limited long-term effectiveness, and further robust evidence is needed to support their use. 19 74 75 76 77 78 79

Dental bleaching as a treatment for enamel demineralization mainly masks or reduces the color difference rather than addressing the underlying issues; therefore, alternative approaches are recommended. 8 84 85 Microabrasion is suitable for shallow lesions as it removes some of the enamel structures, which is not favored in modern dentistry, which emphasizes minimally invasive management techniques. 8 17 87 Resin infiltration is often considered a restorative treatment and tends to be more effective for wider, deeper, or pitted lesions.

Overall, different management approaches and trendy use of lasers for managing demineralization require further studies to evaluate and compare their effectiveness. 8 86 88

## Conclusion

Maintaining the integrity of dental enamel is essential, and prevention plays a key role in this process. Good oral hygiene is the most effective method for preventing and minimizing the occurrence of enamel demineralization and the development of WSLs. In clinical practice, visual inspection remains the preferred diagnostic approach for identifying these lesions. The management of WSLs should be tailored to the individual patient, taking into account factors such as susceptibility, lesion size, depth, and progression, with the standard treatment protocol typically involving facilitating natural mineralization and the application of low-fluoride agents to promote remineralization and restore enamel integrity.
